# Generation of high-affinity, internalizing anti-FGFR2 single-chain variable antibody fragment fused with Fc for targeting gastrointestinal cancers

**DOI:** 10.1371/journal.pone.0192194

**Published:** 2018-02-08

**Authors:** Aleksandra Borek, Aleksandra Sokolowska-Wedzina, Grzegorz Chodaczek, Jacek Otlewski

**Affiliations:** 1 Department of Protein Engineering, Faculty of Biotechnology, University of Wroclaw, Wroclaw, Poland; 2 Wroclaw Research Centre EIT+, Wroclaw, Poland; Imperial College London, UNITED KINGDOM

## Abstract

Fibroblast growth factor receptors (FGFRs) are promising targets for antibody-based cancer therapies, as their substantial overexpression has been found in various tumor cells. Aberrant activation of FGF receptor 2 (FGFR2) signaling through overexpression of FGFR2 and/or its ligands, mutations, or receptor amplification has been reported in multiple cancer types, including gastric, colorectal, endometrial, ovarian, breast and lung cancer. In this paper, we describe application of the phage display technology to produce a panel of high affinity single chain variable antibody fragments (scFvs) against the extracellular ligand-binding domain of FGFR2 (ECD_FGFR2). The binders were selected from the human single chain variable fragment scFv phage display libraries Tomlinson I + J and showed high specificity and binding affinity towards human FGFR2 with nanomolar *K*_D_ values. To improve the affinity of the best binder selected, scFvF7, we reformatted it to a bivalent diabody format, or fused it with the Fc region (scFvF7-Fc). The scFvF7-Fc antibody construct presented the highest affinity for FGFR2, with a *K*_D_ of 0.76 nM, and was selectively internalized into cancer cells overexpressing FGFR2, Snu-16 and NCI-H716. Finally, we prepared a conjugate of scFvF7-Fc with the cytotoxic drug monomethyl-auristatin E (MMAE) and evaluated its cytotoxicity. The conjugate delivered MMAE selectively to FGFR2-positive tumor cells. These results indicate that scFvF7-Fc-vcMMAE is a highly potent molecule for the treatment of cancers with FGFR2 overexpression.

## Introduction

Gastrointestinal cancer is highly prevalent worldwide, with gastric cancer being the second most common cause of cancer mortality [1–3] and 5-year survival rate of approximately 20% [4, 5]. Colorectal cancer is the third most common human malignancy worldwide [6]. Surgical treatment is an effective therapy for gastrointestinal cancer, but unfortunately cannot be performed when the malignancy is detected at advanced stage of development. It is thus of critical importance to identify other means of therapy that would be effective also for treatment of gastrointestinal cancer metastasis and recurrence [1]. Among the more promising therapeutic strategies for comprehensive treatment of gastrointestinal cancer are targeted therapies, including monoclonal antibodies and small-molecule inhibitors [7, 8]. The critical initial step in developing cancer-targeting antibody is identification of specific antigens and generation of antibodies against these antigens with optimal functional characteristics: high-affinity binding and efficient internalization into targeted cells [8].

Fibroblast growth factor receptor 2 (FGFR2) is considered as a potential therapeutic target in multiple types of cancer since its substantial overexpression has been observed in diverse tumor cells and low level of expression on the surface of non-tumor cells [9]. *FGFR2* gene was originally identified as an amplified DNA sequence from the gastric cancer cell line Kato III [10, 11], and subsequent efforts identified FGFR2 amplification in 3% to 10% of primary gastric cancers [12–14]. *FGFR2* gene amplification resulting in FGFR2 overexpression and constitutive activation is also associated with colorectal cancers [15, 16]. High expression and activation of FGFR2 was observed in NCI-H716 colorectal cancer cells, and FGFR2-selective small molecule inhibitors or FGFR2-specific shRNA strongly inhibited cell viability *in vitro*, indicating addiction of NCI-H716 cells to FGFR2 [17].

The association of FGFR2 amplification, overexpression or mutation with gastrointestinal cancers suggests that this receptor could be convenient therapeutic target. Currently, a number of potential FGFR inhibitors are in clinical development. However, the vast majority of them such as dovitinib, nintedanib, or ponatinib are neither highly specific nor selective and inhibit the kinase activity of several other receptors because of the high homology between the kinase domains of VEGFR, PDGFR, and FGFR [18]. Until now, no selective small-molecule FGFR inhibitor has been approved for clinical use [19]. These data confirm that an antibody specific for FGFR2 could be a promising therapeutic agent in the treatment of gastrointestinal cancers associated with FGFR2 overexpression.

To explore FGFR2 as a therapeutic target, we obtained, optimized and fully characterized a panel of high-affinity antibody fragments, specific for human FGFR2. The binding variants were selected from Tomlinson I and J libraries, reformatted to bivalent formats of scFv-Fc or diabody and assayed against human gastric and colorectal cancer lines. We investigated the therapeutic potential of the scFv-Fc anti-FGFR2 antibody by assessing its ability to internalize into live gastrointestinal cancer cells via receptor-mediated endocytosis. To enhance the potency of the FGFR2-targeted therapy, we generated an antibody-drug conjugate (ADC), scFvF7-Fc-vcMMAE, consisting of the scFvF7-Fc antibody fragment chemically conjugated to the cytotoxic agent monomethyl auristatin E (MMAE). MMAE, a synthetic analog of the natural product dolastatin 10 [20], was conjugated to scFvF7-Fc via a peptide linker cleavable by lysosomal enzymes after internalization. Finally, we demonstrated effective *in vitro* growth inhibition of FGFR2-expressing gastric carcinoma (Snu-16) and colorectal carcinoma (NCI-H716) cell lines by the scFvF7-Fc-MMAE conjugate.

## Materials and methods

### Antigen expression and characterization

Extracellular domains (ECD) of FGFR1, FGFR2, FGFR3 and FGFR4 in fusion with the Fc antibody fragment were cloned, expressed and purified as described previously [21]. The Fc fragment allowed efficient single-step purification of the proteins by affinity chromatography on Protein A Sepharose.

The recombinant protein ECD_FGFR2-Fc was used as the molecular target for the selection of a range of specific antibodies from phage display libraries. In addition, ECD_FGFR1-Fc, ECD_FGFR3-Fc, ECD_FGFR4-Fc and Fc fragment alone were used in affinity cross analysis of the selected scFvs.

### Selection from phage display libraries and identification of antibody fragments

For solid surface selection the target protein ECD_FGFR2-Fc was immobilized in wells of a 96-well plate overnight at the concentration of 100 μg/mL. Plates were blocked with 2% skimmed milk powder in PBS (MPBS) for 2 h at room temperature (RT). Before each round of panning phage particles were incubated for 30 min with competitor Fc protein at the concentration of 10^−5^ M, followed by 2-h incubation on 96-well plate (40 min with mixing, 80 min without mixing). The unbound phage was removed by washing 10 times with PBST (PBS containing 0.1% Tween) followed by 10 washes with PBS. Bound phage was eluted with 100 mM triethylamine and neutralized with 1 M Tris-HCl pH 7.2. The eluted phage particles were used for infection of exponentially growing *E*. *coli* TG1 for 30 min at 37°C. Titration of eluted phage, phage amplification and colony picking were performed as described previously [22]. The selection procedure comprised three rounds of panning.

### Monoclonal phage ELISA

Monoclonal ELISA was used for initial screening of scFv clones. Individual bacterial colonies were inoculated into 200 μL of 2× TY/100 μg ampicillin/0.1% glucose in 96-well plates and incubated for 3 h at 37°C with shaking. Expression was induced by addition of 1 mM IPTG and the cultures were grown at 30°C overnight. Antigen was immobilized on Nunc MaxiSorp plates (Thermo Scientific) at the same concentration as for the selection. Bacterial supernatants were added to the immobilized antigen and bound antibody fragments were detected with monoclonal mouse antibody 9E10 (Sigma-Aldrich, St. Louis, MO, USA), followed by anti-mouse IgG horseradish peroxidase immunoglobulin conjugate (Sigma-Aldrich). The assay was developed with TMB soluble substrate (Sigma-Aldrich). The reaction was stopped by addition of 1 M H_2_SO_4_ and the absorbance values were measured at 450 nm.

### Surface plasmon resonance screening of selected clones

The antibody fragments positive in the ELISA assay were further evaluated using surface plasmon resonance (SPR) screening on a BIAcore3000 instrument (GE Healthcare, Little Chalfont, UK). The bacterial supernatants were filtered using 0.22 μm filters and analyzed for ligand binding on CM5 sensor chip coated with covalently immobilized extracellular domain of FGFR2 at about 7,000 RU (high-density sensor chip).

### scFv expression and purification

Recombinant scFv antibody fragments were expressed in *E*. *coli* HB2151 and purified from culture supernatant by affinity chromatography on Protein A Sepharose (GE Healthcare) as described by Villa and colleagues [23] and according to the manufacturer’s instructions. Briefly, supernatant was applied on a column pre-equilibrated with PBS, the unbound fraction was removed and the resin was washed with PBST and PBS buffer. Antibodies were eluted with high pH (100 mM triethylamine), neutralized and dialyzed to PBS. Purified antibody fragments were characterized by SDS-PAGE and size exclusion chromatography on Superdex 75 (GE Healthcare), and fractions containing pure protein of the correct molecular mass were collected.

### Affinity measurements by SPR

Pure antibody fragments were serially diluted and analyzed on a BIAcore3000 instrument for binding to extracellular domain of FGFR2 immobilized on CM5 sensor chip at about 650 RU (low-density sensor chip). Antibodies were injected using the kinject command at a flow of 10 μl/min. For regeneration of the sensor chip surface 10 mM glycine was used. The binding curves of individual clones were analyzed with the BIAevaluation 4.1 software using a 1:1 Langmuir binding model. All measurements were performed in duplicate.

### Cloning and expression of diabody

scFvF7 was reformatted into diabody format by introducing a five-amino acid linker (GGGGS) between VH and VL as described [23]. VH and VL were amplified from scFv plasmid DNA using primer pairs A/C and D/B (Table 1). These two PCR products were assembled in the presence of primer pair A/B (Table 1). The obtained insert was then doubly digested with NcoI/NotI and ligated into NcoI/NotI-digested pIT2 expression vector. The scFv fragments with the linker, forming stable noncovalent homodimers, were expressed and purified in the same way as the original scFv fragments, followed by a final purification step on a Superdex 75 size exclusion chromatography column.

**Table 1:** Primers used for diabody and scFv-Fc cloning.

Symbol	Primer name	Nucleotide sequence
A	LMB3long	5'CAGGAAACAGCTATGACCATGATTA 3'
B	fdseqlong	5'GACGTTAGTAAATGAATTTTCTGTATGAGG 3'
C	DP47link5DPKfo	5'CGTACCGCCACTGGACCCGCTCGAGACGGTGACCAGGGTTCC 3’
D	DPK9link5DP47ba	5'CGAGCGGGTCCAGTGGCGGTACGGACATCCAGATGACCCAGTCT 3'
E	FORscFvSSP1	5'CTCTTCTTCCTGTCAGTAACGACTGGTGTCCACTCCCAGCTGTTGGAGTCTGGGGGAGGC 3'
F	REVscFvKpn2I	5'TACGTCCGGATGCGGCCGCCCGTTTGATTTCC 3 '
G	FORSSPHindII	5'CTCCAAGCTTTGAACCACCATGGAATGGAGCTGGGTCTTTCTCTTCTTCCTGTCAGTAACGACTGG 3'

### Cloning and expression of scFvF7-Fc fusion format

The expression construct of scFvF7 fused to the Fc fragment was prepared in the mammalian expression vector pLEV113-Kpn2I-Fc [21] containing Fc sequence preceded by Kpn2I restriction site. DNA encoding scFvF7 was amplified by two rounds of PCR. The first round introduced a fragment encoding part of the extracellular targeting signal peptide at the 5′ end (primer E, Table 1) and a Kpn2I site at the 3′ end (primer F, Table 1). The second round completed the secretion signal peptide and introduced a HindIII site (primer G, Table 1). Finally, the PCR product was digested with HindIII/Kpn2I and ligated into the HindIII/Kpn2I sites of pLEV113-Kpn2I-Fc. The construct was used for the transfection of CHO-S cells. The cells were grown in suspension medium (PowerCHO-2CD, Lonza) for 6 days. scFv-Fc was purified from supernatant of the suspension culture by affinity chromatography on Protein A Sepharose [21]. The purified protein was analyzed by SDS–PAGE and size exclusion chromatography on Superdex 200 HR 10/30.

### Cell culture

The FGFR2-overexpressing human gastric cell line Snu-16 (no. CRL-5974; American Type Culture Collection, ATCC) and human colorectal cell line NCI-H716 (no. CCL-251; ATCC) were cultured in RPMI-1640 medium (Thermo Fisher Scientific, Waltham, MA, USA). Human osteosarcoma cells U2OS (no. HTB-96; ATCC) were grown in Dulbecco’s modified Eagle’s medium (DMEM) (Invitrogen). All media were supplemented with 10% fetal calf serum (FBS, Invitrogen) and 1% penicillin/streptomycin mix (Gibco). All cells were cultured at 37°C in 5% CO_2_ atmosphere and 95% humidity.

### Immunoblotting

SNU-16, NCI-H716 and U2OS cells were seeded in six-well plates at a density of 0.5 × 10^6^ cells/well. After 24 hours, cells were washed with PBS and lysed with lysis buffer (20 mM Tris-HCl, 150 mM NaCl, 1 mM EDTA, 1% Triton X-100, 1 mM PMSF, protease inhibitor cocktail (Roche)). Total cell lysates were subjected to SDS-PAGE separation and Western blotting. Membranes were probed with anti-FGFR2 rabbit antibody (11835; Cell Signaling Technology) and anti-tubulin mouse antibody (T9026; Sigma-Aldrich), followed by incubation with anti-rabbit and anti-mouse HRP-conjugated secondary antibodies, respectively (Jackson ImmunoResearch). Blots were developed with the ECL reagent (Pierce, Thermo Fisher Scientific) according to the manufacturer’s instructions.

### Confocal microscopy

Antibody fragment scFvF7-Fc was labeled with DyLight 550 (Pierce, Thermo Fisher Scientific) following the manufacturer’s instructions. Snu-16 and NCI-H716 cells (3 × 10^5^ to 5 × 10^5^) were washed in RPMI 1640–10% FBS and incubated on poly-D-lysine (Sigma-Aldrich)-treated coverslips at 37°C for up to 60 min. DyLight550-scFvF7-Fc was added to 50 μg/mL and incubated for 15 min at 37°C. The cells were then fixed with 4% paraformaldehyde for 15 min, permeabilized with 0.5% Triton X-100 (Sigma-Aldrich) for 10 min and blocked with blocking buffer (1% BSA, 10% normal goat serum, 0.3 M glycine, 0.1% Tween in PBS) for 30 min. The cells were further incubated with rabbit anti-early endosomal antigen 1 (EEA1) antibodies (BD Biosciences Transduction Laboratories, Lexington, KY) and stained with goat anti-rabbit Alexa 488-conjugated antibodies (ab150077, Abcam, Cambridge, UK)). Nucleus staining was performed with DAPI reagent (Thermo Fisher Scientific). The cells on a coverslip were mounted with ProLong Gold anti-fade mounting medium (Thermo Fisher Scientific) and observed with a Cell Observer SD confocal system (Zeiss, Oberkochen, Germany) equipped with an EMCCD QImaging Rolera EM-C2 camera and a 63× oil immersion objective. Images were analyzed with Fiji software (NIH, Bethesda, MD, USA).

### Conjugate preparation

scFvF7-Fc was conjugated via its cysteine residues to vcMMAE as previously described [24]. In order to reduce the interchain disulfide bond, scFvF7-Fc was treated with 2.5 equivalents of tris(2-carboxyethyl)phosphine (TCEP) for 3 h at 37°C and then conjugated to vcMMAE in borate buffer (25 mM sodium borate, pH 8.0, 25 mM NaCl, 1 mM EDTA). scFvF7-Fc-vcMMAE was purified through overnight dialysis against PBS.

### Cytotoxicity assay

SNU-16, NCI-H716 and U2OS cells were seeded at a density of 5000 cells per well in a 96-well culture plate and treated with vcMMAE, scFvF7-Fc, or antibody-vcMMAE conjugate scFvF7-Fc-MMAE for 96 hours at concentrations ranging from 0.001 to 1000 nM. Control cells were treated with PBS only. Cell proliferation was determined using AlamarBlue Proliferation Assay Kit (Thermo Fisher Scientific) according to the manufacturer’s instructions. The concentrations required to inhibit cell growth by 50% (IC_50_—half maximal inhibitory concentration) were calculated using OriginPro8

## Results

### Selection from Tomlinson I + J libraries

**Fig 1:**
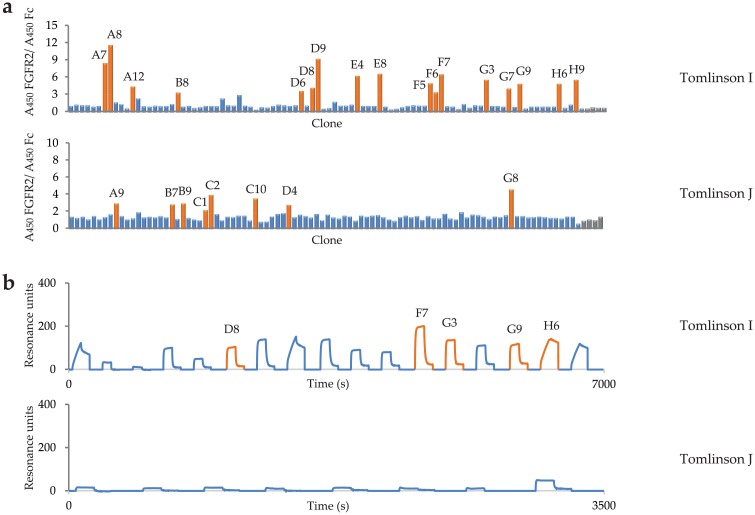
Binding specificities of anti-FGFR2 scFv clones selected from Tomlinson I and J libraries. (a) ELISA analysis of randomly picked clones after 3 rounds of panning against extracellular domain of FGFR2-Fc. Bacterial supernatants containing soluble antibody fragments were applied onto the target protein immobilized on the plastic surface of 96-well plate and separately onto immobilized Fc protein used as a negative control. Selected clones are named according to 96-well plate positions. (b) Surface Plasmon Resonance screening of clones showing the highest absorbance in ELISA assay. scFv clones with different sequences are highlighted.

**Fig 2:**
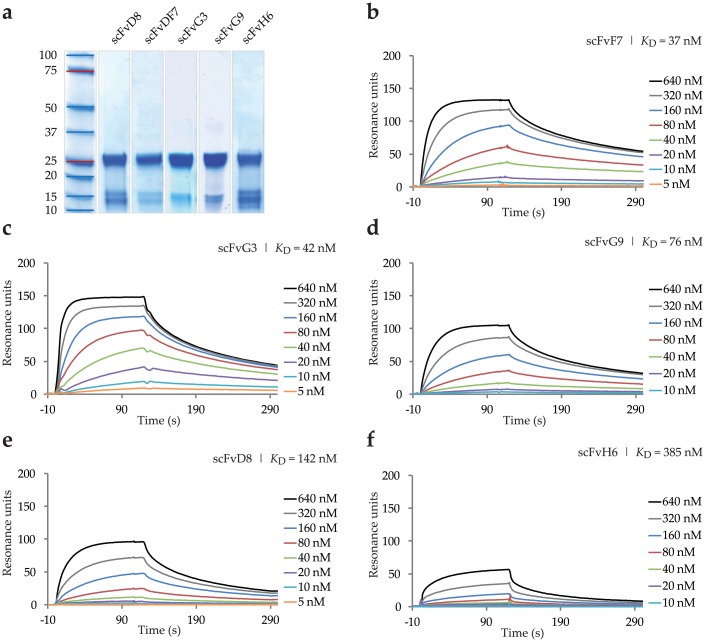
Purification of scFv variants and their binding to FGFR2. (a) Coomassie blue-stained SDS-PAGE profiles of scFvs obtained under reducing conditions. (b-f) Binding curves for five scFv clones against FGFR2. Different concentrations of purified proteins were injected on CM5 sensor chip coated with about 650 RU of mammalian cell-derived FGFR2-Fc protein. BIAevaluation 4.1 software was used for calculation of kinetic constants.

scFv antibodies specific for FGFR2 were selected from the human Tomlinson I + J libraries by phage display. After three rounds of panning we performed series of monoclonal supernatant ELISA assays of selected clones against ECD_FGFR2-Fc and Fc in parallel, to exclude clones specific for Fc fragment, and identified 31 clones positive for FGFR2 (Fig 1a). The ELISA-positive clones were then screened by SPR real-time interaction analysis on a high-density antigen-coated sensor chip, using a BIAcore instrument (Fig 2b). A strong interaction between FGFR2 and 15 individual clones was confirmed. These clones were further subjected to nucleotide sequencing. Based on the binding specificity and predicted amino acid sequences of the heavy and light chain variable regions CDR2 and CDR3, we selected five unique variants of scFvs specific for FGFR2 (Fig 1b, Table 2). The scFvs were then expressed in HB2151 bacteria and purified on a Protein A resin. Purified scFvs were analyzed by gel electrophoresis under reducing conditions, followed by Coomassie blue staining (Fig 2a).

**Table 2:** Amino acid sequences of randomized positions of the CDR2 and CDR3 regions of five selected scFv fragments. Antibody residues are numbered according to Tomlinson et al. [25] and Cox et al.[26].

Antibody name	CDR2 heavy chain	CDR3 heavy chain	CDR2 light chain	CDR3 light chain
	Positions: 50, 52, 52a, 53, 55, 56, 58	Positions: 95, 96, 97, 97	Positions: 50, 53	Positions: 91, 92, 93, 94, 96
scFvD8	G Y D N T A A	G G S S	N T	N G Y T S
scFvF7	A S D T S A G	S S G S	A N	T G Y S T
scFvG3	S T D S G G G	S Y Y A	G T	Y S Y S Y
scFvG9	S S D A S A G	S A S S	D S	N G Y S S
scFvH6	S G D S S T A	S A G A	S A	T G Y T T

To determine the kinetic binding parameters of the scFvs, monomeric fractions of purified binders were used for BIAcore affinity measurements on a CM5 sensor chip coated with about 650 RU of ECD_FGFR2-Fc protein. The selected scFvs displayed high binding affinity to human ECD_FGFR2 with *K*_D_ values in the nanomolar range as shown in Fig 2b–2f (apparent kinetic rate constants are reported in Table 3). scFvF7, scFvG3 and scFvG9 exhibited the best parameters, with the dissociation constant of 37 nM, 42 nM and 76 nM, respectively (Fig 2c–2e).

**Table 3:** Kinetic parameters of anti-FGFR2 scFv fragments.

Antibody name	*K*_D_ (M)^a^	*k*_on_ (1/Ms)^a^	*k*_off_ (1/s)^a^
scFvD8	1.4 2x 10^−7^	5.39 x 10^4^	7.65 x 10^−3^
scFvF7	3.70 x 10^−8^	1.06 x 10^5^	3.99 x 10^−3^
scFvG3	5.93 x 10^−8^	9.86 x 10^4^	5.85 x 10^−3^
scFvG9	7.60 x 10^−8^	7.12 x 10^4^	5.43 x 10^−3^
scFvH6	3.85 x 10^−7^	2.53 x 10^4^	9.75 x 10^−3^

^a^ Measured on the BIAcore3000 instrument. Kinetic constants were calculated with the BIA evaluation 4.1 software. Fitting error for global K_D_ fit was below 2,5%.

**Fig 3:**
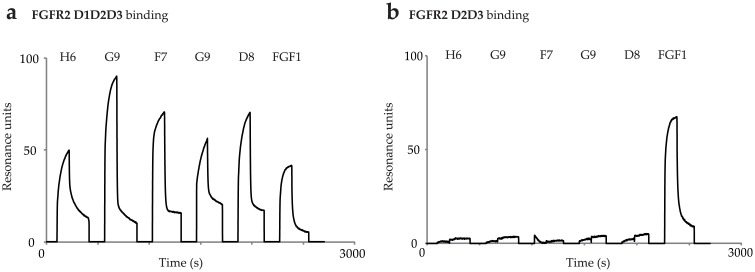
Mapping of scFv binding site. Binding of scFvs to (a) ECD_FGFR2-D1D2D3-Fc, (b) ECD_FGFR2-D2D3-Fc. Natural ligand FGF1 was used as a positive control.

For further characterization we determined the receptor binding sites of antibody fragments. We analyzed binding of scFvs to full length extracellular domain of FGFR2 composed of D1, D2, and D3 domains (ECD_FGFR2-D1D2D3-Fc) and to a shorter variant composed of D2 and D3 domains (ECD_FGFR2-D2D3-Fc). All of scFv molecules showed binding affinity towards full FGFR2-D1D2D3 (Fig 3a) and no binding was observed towards FGFR2-D2D3 (Fig 3b). Using this approach we confirmed that antibody fragments recognized D1 domain of FGFR2. However, they could bind to an epitope that is partly located on D1 domain, and partly on another part of the molecule. Fibroblast growth factor 1 (FGF1), a natural ligand of all FGFR isoforms, which binds to D2-D3 region of FGFR2 was used as a positive control. As expected, FGF1 bound to both FGFR2 variants tested (Fig 3).

**Fig 4:**
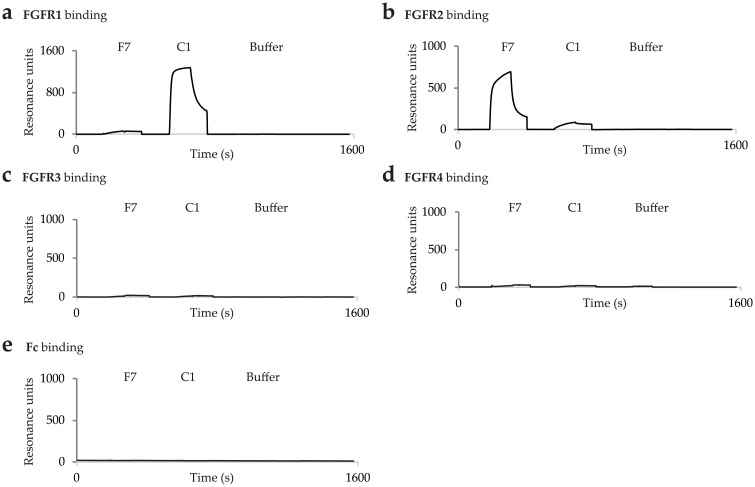
SPR binding profiles of anti-FGFR2 scFvF7 and anti-FGFR1 scFvC1 to FGFRs. Binding of scFvs to (a) ECD_FGFR1-Fc, (b) ECD_FGFR2-Fc, (c) ECD_FGFR3-Fc, (d) ECD_FGFR4-Fc and (e) Fc. Clone scFvC1 was selected against ECD_FGFR1 [27] and was used as a negative control.

Furthermore, we tested the specificity of the strongest binder scFvF7 towards other FGF receptors. We found that scFvF7 displayed no cross reactivity with FGFR1, 3 and 4, and no affinity to the Fc fragment, which was confirmed by SPR analysis using high-density sensor chips coated with ECD_FGFR1-Fc, ECD_FGFR3-Fc, ECD_FGFR4-Fc, and Fc (Fig 4).

### Reformatting of selected scFv into bivalent formats

**Fig 5:**
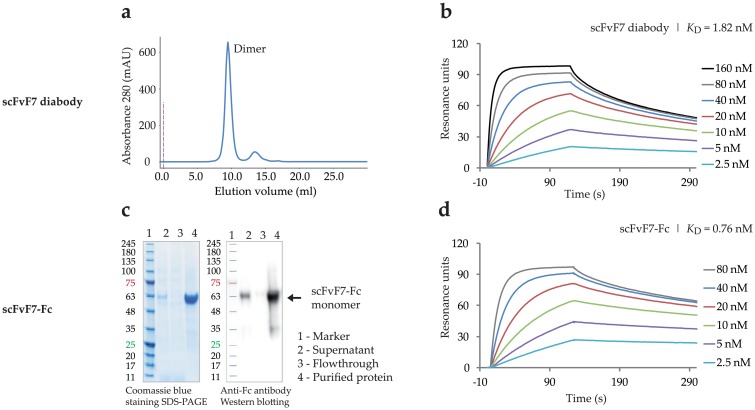
Characteristics of scFvF7 bivalent formats. (a) SEC gel-filtration profile of scFvF7 diabody. (c) SDS–PAGE electrophoresis and Western blotting of scFvF7-Fc fragment. Binding curves against FGFR2 for (b) scFvF7 diabody and (d) scFvF7-Fc. scFvF7 diabody and scFvF7-Fc in different concentrations were injected on CM5 sensor chip coated with about 650 RU of ECD_FGFR2-Fc. BIAevaluation 4.1 software was used for calculation of kinetic constants.

To improve functional recognition of FGFR2, bivalent formats of scFv, diabody and scFv-Fc, were constructed for clone scFvF7 showing the highest affinity for FGFR2. A diabody is a non-covalent homodimer formed by two scFv molecules, whereas the scFv-Fc fusion forms disulfide-linked homodimers. The diabody was expressed in a bacterial system, whereas the scFv-Fc fusion protein was expressed in CHO-S cells using pLEV113 vector and transient gene expression. Both bivalent formats were purified by a single-step affinity chromatography on Protein A Sepharose, as described in Materials and Methods. The scFvF7 diabody was characterized by size exclusion chromatography on Superdex 75 (Fig 5a), and scFvF7-Fc was analyzed by SDS–PAGE under reducing conditions, followed by Coomassie blue staining and Western blotting with anti-Fc antibodies (Fig 5c).

The binding properties of the reformatted molecules were confirmed by BIAcore analysis using a low-density sensor chip coated with ECD_FGFR2-Fc. As expected, both the scFvF7 diabody and scFvF7-Fc exhibited improved binding to FGFR2 compared with scFvF7. Their SPR binding profiles are presented in Fig 5b and 5d and the kinetic constants are reported in Table 4. The bivalent formats of scFvF7 showed dissociation constants of 1.82 nM (diabody) and 0.76 nM (Fc-fusion), representing, respectively, a 20- and 48-fold improvement of the FGFR2 binding affinity over the parental scFvF7.

**Table 4:** Kinetic constants of bivalent formats of scFvF7.

	*K*_D_ (M)^a^	*k*_on_ (1/Ms)^a^	*k*_off_ (1/s)^a^
scFvF7	3.70 x 10^−8^	1.06 x 10^5^	3.99 x 10^−3^
scFvF7 diabody	1.82 x 10^−9^	1.34 x 10^6^	2.50 x 10^−3^
scFvF7-Fc	0.76 x 10^−9^	1.61 x 10^6^	1.22 x 10^−3^

^a^ Measured on the BIAcore3000 instrument. Kinetic constants were calculated with the BIA evaluation 4.1 software. Fitting error for global K_D_ fit was below 2,5%.

### Internalization of anti-FGFR2 scFvF7 bivalent formats

To evaluate the potential of the engineered scFvF7 bivalent formats to deliver agents into tumor cells expressing FGFR2, internalization of DyLight550-labeled scFvF7 diabody and scFvF7-Fc into Snu-16 and NCI-H716 cells was monitored by confocal microscopy. U2OS cells not expressing FGFR2, as confirmed by Western blot analysis using anti-FGFR2 antibodies (S1 Fig), served as a negative control.

**Fig 6:**
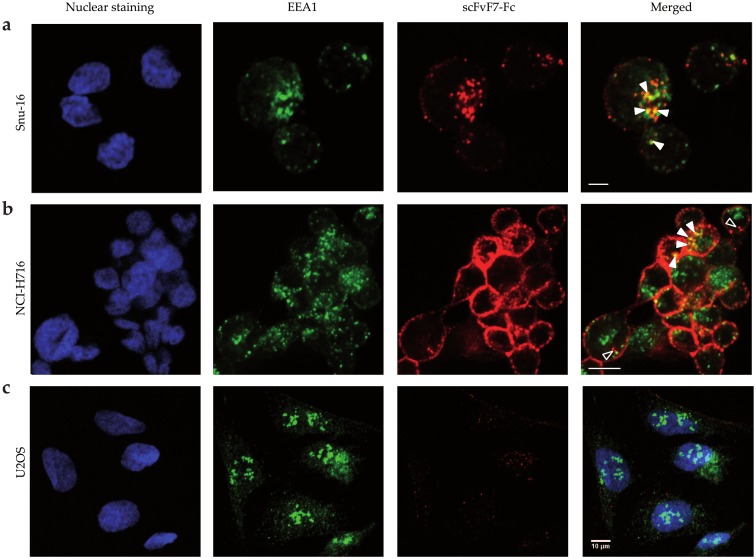
Localization of scFvF7-Fc in FGFR2-positive and FGFR2-negative cells. Fluorescence was detected in fixed and permeabilized (a) Snu-16, (b) NCI-H716, (c) U2OS cells. scFvF7-Fc was stained red with DyLight550. EEA1 was labeled with anti-EEA1 antibody detected with Alexa Fluor 488–conjugated secondary antibody (green staining). Nuclei were stained with DAPI (blue). The scale bar in all panels represents 10 μm.

The fluorescently labeled scFvF7 diabody or scFvF7-Fc was incubated with Snu-16, NCI-H716 and U2OS cells for 15 minutes at 37°C and then the cells were inspected for fluorescence. Low level of scFvF7 diabody internalization was found (data not shown), whereas scFvF7-Fc was efficiently internalized by FGFR2-expressing cells (Snu-16 and NCI-H716) (Fig 6a and 6b) as judged by the partial colocalization of the DyLight550 signal among cytoplasmic endosomal granules visualized with anti-EEA1 antibody binding a protein associated with early/sorting endosomes (solid white arrowheads indicate colocalizing pixels) [28]. Internalization was more efficient in case of Snu-16 cells as majority of DyLight550 signal disappeared from the cell membrane, while in NCI-H716 cells DyLight550 was present in the cytoplasm (open and solid arrowheads) but also clustered in the cell membrane. This suggests that scFvF7-Fc can be internalized at different rates depending on the cell line to early endosomes from which it is transported to other intracellular compartments. Only low level of plasma membrane binding and internalization of labeled scFvF7-Fc was observed in the FGFR2-negative cell line U2OS (Fig 6c). These results confirm that scFvF7-Fc is internalized in a receptor-dependent manner and therefore we used this molecule to the subsequent part of the study.

### *In vitro* cytotoxicity of scFvF7-Fc-vcMMAE

**Fig 7:**
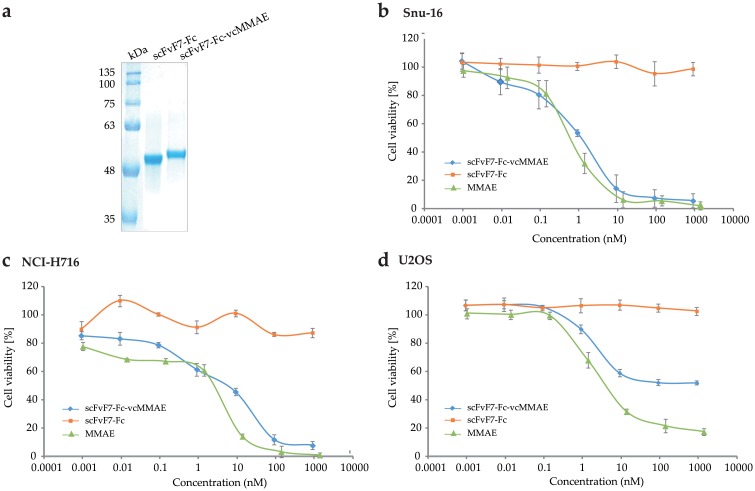
Characterization of the scFvF7-Fc-vcMMAE conjugate. (a) SDS-PAGE analysis of scFvF7-Fc and scFvF7-Fc-vcMMAE under reducing conditions. (b-d) Cytotoxicity of scFvF7-Fc-vcMMAE, scFvF7-Fc and MMAE on (b) Snu-16, (c) NCI-H716, and (d) U2OS cells. Cell viability was determined by AlamarBlue Proliferation Assay Kit. Data show average values of triplicate wells ± SD from a representative experiment of two that were performed.

Since confocal microscopy confirmed that scFvF7-Fc was effectively internalized by cancer cell lines with FGFR2 overexpression, we conjugated it with the cytotoxic drug vcMMAE in order to produce an antibody-drug conjugate. The conjugate, named scFvF7-Fc-vcMMAE, was homogeneous, as shown by SDS-PAGE analysis (Fig 7a).

The cytotoxicity and selectivity of scFvF7-Fc-vcMMAE was evaluated on FGFR2-positive (Snu-16 and NCI-H716) and FGFR2-negative (U2OS) cell lines. After 96 hours of continuous exposure to the conjugate, cytotoxicity was assessed by AlamarBlue assay. scFvF7-Fc-vcMMAE exhibited substantial cytotoxicity against the FGFR2-positive cell lines: Snu-16 and NCI-H716 with the half-maximal inhibitory (IC_50_) values of 0.89 and 7.7 nM, respectively (Fig 7b and 7c). In contrast, the FGFR2-negative line U2OS showed limited sensitivity to scFvF7-Fc-vcMMAE, poorly dependent on the conjugate concentration outside the 0.1–10 nM range and the IC_50_ in excess of 1000 nM (Fig 7d). None of the cell lines tested were affected by unconjugated scFvF7-Fc at concentrations up to 1000 nM (Fig 7b–7d), but all three were sensitive to MMAE with the IC_50_ values of 0.66, 3.12, and 4.93 nM, respectively (Fig 7b–7d).

## Discussion

The number of monoclonal antibodies (mAbs) for cancer treatment that have been approved or are in clinical development has been steadily increasing over recent years. The success of mAbs such as trastuzumab and cetuximab as cancer therapeutics has generated great interest in the development of specific antibodies binding to new receptor tyrosine kinase (RTKs) targets or to novel epitopes of known targets [29]. As dysregulated FGFR signaling has been associated with the development of numerous human malignancies [30], new therapeutic antibodies targeting individual FGFRs could have broad clinical applications.

FGFR2 amplification is detected in approximately 3% to 10% of gastric cancers and correlates with a poor prognosis for patients [12–14]. Patients with FGFR2 amplification have significantly shorter overall survival time than those without FGFR2 amplification [31]. Amplification of the *FGFR2* gene is also associated with colorectal cancers [15, 16]. Previously, a multikinase small-molecule inhibitor, AZD2171, was reported to have antitumor activity against gastric cancer xenografts overexpressing FGFR2 [32]. However, due to the broad spectrum of targets affected by AZD2171, its *in vivo* growth inhibitory activity could also be attributed to the suppression of other kinases in addition to FGFR2. According to the World Health Organization, gastric cancer accounts for nearly one in ten of all deaths of cancer patients [33]. Mean survival for patients with stage IV metastatic gastric and colorectal cancer is only 10 [34] and 12 months [35], respectively. Given this, there is an urgent need to improve gastrointestinal cancer therapy.

In this study we report the selection by phage display and characterization of human antibody fragments specific for the extracellular domain of human FGFR2. To obtain a valuable recognition tool for ECD_FGFR2 we used commercially available Tomlinson I and J libraries as the source of high-affinity binders. By optimizing a well-established procedure of selection [23] scFv antibody fragments with favorable binding profile towards the antigen protein were obtained with the best scFvF7 fragment showing the dissociation constant *K*_D_ of 3.7 x 10^−8^ M. However, scFvF7, which has an approximate molecular weight of 27 kDa, has limited applicability due to its rapid *in vivo* clearance and a consequent short half-life [36], and a lack of immunologic effector functions contributed by the Fc region of IgG [37]. To overcome these limitations, we generated bivalent formats: scFvF7 diabody and a fully human scFvF7-Fc antibody fragment by fusing scFvF7 to a human IgG1 Fc region. The resulting scFvF7 diabody and scFvF7-Fc showed dissociation constants of 1.82 × 10^−9^ M and 0.76 × 10^−9^ M, respectively in their interaction with the antigen, that is 20- and 48-fold less than monomeric scFvF7.

Precise targeting of cancer cells is the crucial aspect of successful antitumor therapies. Specific ligands should not only mark the cancerous cell, but also be specifically internalized in order to act intracellularly without affecting the surrounding healthy cells [38]. To confirm the biological activity and to investigate the specific cell-penetrating ability of the scFvF7 diabody and scFvF7-Fc, confocal microscopy was performed on FGFR2-overexpressing cancer lines. It demonstrated that scFvF7-Fc was effectively internalized by Snu-16 and NCI-H716 cells and therefore we performed conjugation with the cytotoxic compound vcMMAE in order to produce an active antibody-drug conjugate. scFvF7-Fc-vcMMAE showed good selective cytotoxic activity towards cells with FGFR2 overexpression. The IC_50_ values for the tested FGFR2-positive cells were within the nanomolar range (0.89 nM for Snu-16 cells and 7.7 nM for NCI-H716 cells). In contrast, the cytotoxicity towards FGFR2-negative cells was much lower, confirming high specificity of the conjugate. Additionally, we also observed no substantial difference between cytotoxicity of the drug itself and the scFvF7-Fc-drug conjugate against cells presented in the Fig 7b–7d. The IC_50_ of MMAE for FGFR2-positive and FGFR2-negative cells were ranged from 0.66 nM to 4.93 nM and were lower in comparison to those obtained for scFvF7-Fc-drug conjugate. MMAE exhibits non-specific toxicity, as a result of targeting all rapidly dividing cells. Since MMAE is hydrophobic, it can easily diffuse out of the target cell and mediate the killing of nearby cells by so called bystander [39, 40].

Antibody–drug conjugate BAY 1187982 developed by Sommer and colleagues [41], similarly to the scFvF7-Fc-MMAE, targets FGFR2 receptor. This ADC consists of a fully human FGFR2 monoclonal antibody conjugated to an auristatin W derivative. BAY 1187982 exhibited high cytotoxicity against Snu-16 and NCI-H716 cells with IC_50_ values of 0.37 nM and 0.43 nM, respectively. Based on these data, we can see that BAY 1187982 showed similar effect on Snu-16 cells when compared to the scFvF7-Fc-vcMMAE conjugate, as evidenced by the IC_50_ of 0.37 nM (BAY 1187982) and 0.89 nM (scFvF7-Fc-vcMMAE) and higher cytotoxic effect against NCI-H716 cells (IC_50_ 0.37 nM) than scFvF7-Fc-MMAE (IC_50_ 7.7 nM). BAY conjugate 1187982 also reduces the viability of control cells with IC_50_ of 250 nM. In contrast, the U2OS cells, which was used as a negative control in our tests showed limited sensitivity to scFvF7-Fc-vcMMAE (IC_50_ > 1000 nM).

To summarize, we have generated a high-affinity scFvF7-Fc format from the scFvF7 antibody fragment specific for the extracellular domain of FGFR2, which undergoes rapid internalization into cancer cells upon binding to FGFR2. We believe that the scFvF7-Fc antibody represents an attractive tool for use in therapeutic applications associated with pathological overexpression of human FGFR2.

## Supporting information

S1 Fig FGFR2 expression at the protein level. Total cell lysates separated by SDS-PAGE, transferred to the membrane, probed with (a) FGFR2 specific antibodies and visualized with chemiluminescent substrate. Equal loading was confirmed by antibodies specific for tubulin (b).
(PDF)
