# Supplementary material for: Generation of high-affinity, internalizing anti-FGFR2 single-chain variable antibody fragment fused with Fc for targeting gastrointestinal cancers
Source: PLoS One. 2018 Feb 8;13(2):e0192194. doi: 10.1371/journal.pone.0192194 (PMC5805272; doi:10.1371/journal.pone.0192194)
Supplement: S1 Fig — Total cell lysates separated by SDS-PAGE, transferred to the membrane, probed with (a) FGFR2 specific antibodies and visualized with chemiluminescent substrate. Equal loading was confirmed by antibodies specific for tubulin (b). (PDF) [file pone.0192194.s001.pdf]

SUPPORTING INFORMATION

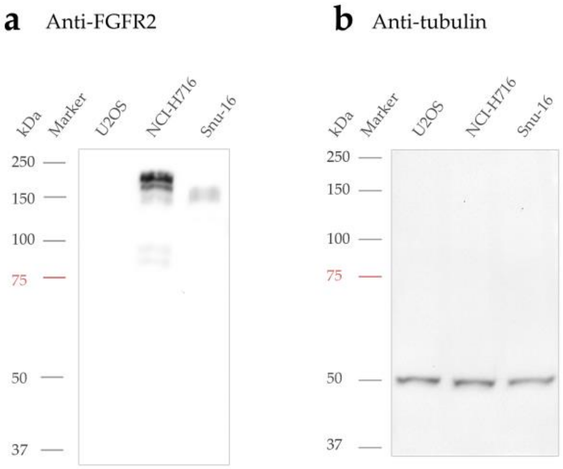

S1 Fig. FGFR2 expression at the protein level. Total cell lysates separated by SDS-PAGE, transferred to the membrane, probed with (a) FGFR2 specific antibodies and visualized with chemiluminescent substrate. Equal loading was confirmed by antibodies specific for tubulin (b).
